# Quality of life after fragility fracture in the Russian Federation: results from the Russian arm of the International Cost and Utility Related to Osteoporotic Fractures Study (ICUROS)

**DOI:** 10.1007/s11657-020-0699-6

**Published:** 2020-03-02

**Authors:** Olga Lesnyak, Axel Svedbom, Ksenia Belova, Olga Dobrovolskaya, Olga Ershova, Georgij Golubev, Vyacheslav Grebenshikov, Sergej Ivanov, Alexander Kochish, Larissa Menshikova, Oxana Nikitinskaya, Radik Nurligayanov, Alexander Solodovnikov, Natalia Toroptsova, Julia Varavko, Eugenij Zotkin, Fredrik Borgstrom, John A Kanis

**Affiliations:** 1 North West Mechnikov State Medical University, St. Petersburg, Russia; 2 Mapi, Stockholm, Sweden; 3 Yaroslavl State Medical University, Yaroslavl, Russia; 4 V.A. Nasonova Research Institute of Rheumatology named after V.A. Nasonova, Moscow, Russia; 5 Rostov-on-Don State Medical University, Rostov-on-Don, Russia; 6 The L.G. Sokolov Memorial Hospital №122, St. Petersburg, Russia; 7 Vreden Russian Research Institute of Traumatology and Orthopedics, St. Petersburg, Russia; 8 Irkutsk State Medical Academy of Postgraduate Training, Irkutsk, Russia; 9 Research Institute of Rheumatology named after V.A. Nasonova, Moscow, Russia; 10 City clinical hospital №21, Ufa, Russia; 11 Ural State Medical University, Yekaterinburg, Russia; 12 Irkutsk State Medical University, Irkutsk, Russia; 13 LIME/MMC, Karolinska Institutet, Stockholm, Sweden; 14 Centre for Metabolic Bone Diseases, University of Sheffield Medical School, Sheffield, UK and Mary McKillop Health Institute, Australian Catholic University, Melbourne, Australia

**Keywords:** Hip fracture, Ankle fracture, Vertebral fracture, Distal forearm fracture, Humerus fracture, ICUROS, Osteoporosis, Quality of life

## Abstract

***Summary*:**

Changes in health-related quality of life (QoL) due to hip, humeral, ankle, spine, and distal forearm fracture were measured in Russian adults age 50 years or more over the first 18 months after fracture. The accumulated mean QoL loss after hip fracture was 0.5 and significantly greater than after fracture of the distal forearm (0.13), spine (0.21), proximal humerus (0.26), and ankle (0.27).

**Introduction:**

Data on QoL following osteoporotic fractures in Russia are scarce. The present study evaluated the impact of hip, vertebral, proximal humerus, distal forearm, and ankle fracture up to 18 months after fracture from the Russian arm of the International Costs and Utilities Related to Osteoporotic Fractures Study.

**Methods:**

Individuals age ≥ 50 years with low-energy-induced humeral, hip, clinical vertebral, ankle, or distal forearm fracture were enrolled. After a recall of pre-fracture status, HRQoL was prospectively collected over 18 months of follow-up using EQ-5D-3L. Multivariate regression analysis was used to identify determinants of QALYs loss.

**Results:**

At 2 weeks, patients with hip fracture (*n* = 223) reported the lowest mean health state utility value (HSUV) compared with other fracture sites. Thereafter, utility values increased but remained significantly lower than before fracture. For spine (*n* = 183), humerus (*n* = 166), and ankle fractures (*n* = 214), there was a similar pattern of disutility with a nadir within 2 weeks and a progressive recovery thereafter. The accumulated mean QoL loss after hip fracture was 0.5 and significantly greater than after fracture of the distal forearm (0.13), spine (0.21), proximal humerus (0.26), and ankle (0.27). Substantial impairment in self-care and usual activities immediately after fracture were important predictors of recovery across at all fracture sites.

**Conclusions:**

Fractures of the hip, vertebral, distal forearm, ankle, and proximal humerus incur substantial loss of QoL in Russia. The utility values derived from this study can be used in future economic evaluations.

## Introduction

Osteoporosis is a skeletal disorder characterized by compromised bone strength predisposing to an increased risk of fracture. Bone strength primarily reflects the integration of bone density and bone quality [1, 2]. Osteoporosis results in fractures that impose a considerable financial burden on health services due to reduced mobility, hospitalization, and nursing home requirements [3, 4]. Worldwide, osteoporosis causes more than 8.9 million fractures annually, equating with a new osteoporotic fracture every 3 s [5]. In the Western world, 1 in 3 women over age 50 years will experience osteoporotic fractures, as will 1 in 5 men aged over 50 years [6].

Loss of quality of life (QoL) after fragility fracture is significant, although, until recently, the numbers of studies and patients included were limited and the length of follow-up relatively short [7]. To overcome these issues and to estimate the costs and quality of life related to fractures in a number of countries across the world, the International Osteoporosis Foundation initiated the International Costs and Utilities Related to Osteoporotic Fractures Study (ICUROS) in 2007. Results of the ICUROS [8, 9] showed that fragility hip, vertebral, and distal forearm fractures resulted in substantial QoL loss directly after fracture. While QoL improved with time, 18 months after fracture, mean health state utility values (HSUVs) were lower than before the fracture in patients with hip fracture (0.66 vs. 0.77 *p* < 0.001) and vertebral fracture (0.70 vs.0.83 *p* < 0.001) [8].

Relatively little is known about the consequences of osteoporosis in Russia. The hip fracture rates lie in between the high rates reported in Scandinavia and the low rates reported in Latin America, and approximate those found in Australia and the Netherlands. Similarly, hip fracture probabilities are relatively low and similar to those reported for Hungary [10, 11]. By contrast, probabilities of a major fracture are much higher due to the unexpectedly high rates of forearm and humeral fractures in Russia [12]. There are few data on the consequences of fracture on QoL in Russia. Differences in accumulated QoL losses among countries have been reported but summary data only provided. Compared with Russia, the accumulated QoL losses over 18 months were consistently higher in Italy and Lithuania and lower in Austria for all three fracture types [8]. The objectives of the present study were to estimate QoL changes for patients with hip, vertebral, distal forearm, humeral, and ankle fracture in Russia and to identify predictors of recovery at 18 month after fracture.

## Methods

### Study design and population

The ICUROS was a multinational prospective observational study, the details of which have been previously described [9]. In short, the study enrolled patients with fragility fractures age 50 years and over. 1) Patients residing in long-term care prior to the fracture were excluded; 2) patients with cognitive impairment were excluded; and 3) patients who sustained a new fracture during follow-up were excluded. Patients were interviewed within 14 days after the first health care contact for the fracture and were followed for 18 months using structured questionnaires. For the Russian component of ICUROS, consecutive patients fulfilling the inclusion criteria were recruited at university-related city hospitals in different parts of Russia: Yekaterinburg, St Petersburg (2 centers), Ufa, Moscow (2 centers), Yaroslavl, Irkutsk, Rostov-on-Don.

Questionnaires used were translated into Russian. Patients with pathological fracture (e.g., cancer) or multiple fractures were not eligible. Inclusion criteria comprised patients who had sustained a humeral, hip, clinical vertebral, ankle, or distal forearm fracture, capable of answering the questionnaires and giving their informed consent. All fractures were confirmed by X-ray examination. Patients who sustained a subsequent fracture or died during the follow-up were withdrawn.

The study was conducted in accordance with the declaration of Helsinki, informed consent was obtained from all participants, and the study was approved by the Research Ethics Committee of Ural State Medical University. Patients could withdraw from the study at any time on their own request.

### Study data

The study included five assessments. The baseline assessment corresponded to the immediate pre-fracture period; data for this phase were gathered by recall within 2 weeks of the fracture event. At the same time, data relating to the fracture were collected. Subsequent assessments took place at 4, 12, and 18 months after fracture.

Patient characteristics recorded at baseline included age (years at time of fracture), sex (male/female), presence of previous fractures the last 5 years (yes/no), living arrangements (alone, with spouse, with son/daughter, with friend/relative), employment before fracture (yes/no), proportion of full-time employment (0–100%), reason for not being employed (old-age pensioner, disabled pensioner), education (primary school, secondary school, university education, and professional diploma), and income (low, middle, high).

Changes in health related QoL were assessed using the Russian version of the EQ-5D-3L [13]. The instrument measures five dimensions: mobility, self-care, usual activities, pain/discomfort, and anxiety/depression. Each dimension measures three levels of severity: no problem, some problems, and major problems, giving 243 (35) possible health state combinations. The UK value set of preference weights was used to calculate health utilities [14] as recommended by the EuroQoL group in the absence of country-specific value sets [15]. The scores were anchored at 1 (full health) and 0 (dead) with values below 0 denoting health states worse than death.

In addition to the HSUV estimates before fracture, within 2 weeks after the fracture, and at 4, 12, and 18 months after fracture, accumulated QoL loss, and QoL multipliers were estimated for the time periods 0–6 months, 0–12 months, and 12–18 months, after fracture. Both measures were derived using actual and baseline QoL (assuming no change in QoL had the fracture not occurred). The actual QoL development was derived using linear interpolation between the observed health state utility values (HSUV). The hypothetical QoL development assumes that QoL would have remained at the pre-fracture level had the facture not occurred. Accumulated QoL loss was estimated as the difference between the areas under the curves of the actual and hypothetical QoL trajectories over the relevant time periods. QoL multipliers were estimated as the ratio between the areas under the curves of the actual and hypothetical QoL trajectories over the relevant time periods [8].

Full recovery after fracture was defined as a having a HSUV at 18 months equal or greater than the pre-fracture recall HSUV. Severe impairment in an EQ-5D dimension immediately after fracture was defined as a patient reporting level 3 (“severe problem”) in the relevant dimension at enrolment (within 14 days of fracture).

### Statistical analysis

For baseline characteristics, comparisons among groups were conducted using *t* tests, *F*-tests, or chi-square tests as appropriate. Comparisons of HSUV between fracture sites were conducted using two sample *t* tests. Parametric tests were implemented by virtue of the central limit theorem. All tests were two-tailed with a significance level of 5%.

Given that QoL multipliers are estimated using ratios and the underlying data comprise both negative values and zeros, arithmetic mean estimates of QoL multipliers may be biased. Therefore, bootstrapping was implemented for the relevant time periods (0–6 months, 0–12 months, and 12–18 months after fracture) and 95% confidence intervals (CIs) were derived using the percentile method as described in Svedbom et al. [8].

We estimated the crude and standardized difference in full-recovery at 18 months after fracture stratified by severe impairment in each EQ-5D dimension and fracture type. We derived standardized differences in probability of full recovery using marginal structural binomial regression models [16] adjusted for age at fracture, sex, and EQ-VAS prior to fracture. In these analyses, we excluded patients whose QoL prior to fracture were in the lowest quartile for each fracture type given that patients with substantial impairment prior to fracture may have a high likelihood to recover pre-fracture QoL, even though they report severe impairment directly after fracture.

All analyses were implemented in STATA 14.1.

## Results

### Study population

A total of 1222 subjects fulfilled the inclusion criteria and underwent the initial interview within 2 weeks after fracture. Among those, 28 (2.2%) sustained another fracture and were therefore excluded during follow-up, and 17 (1.3%) died of whom 7 had sustained a hip fracture. Among the remaining 1177 patients, 40 (3.6%) were lost to follow-up at month 4, 9 patients (0.8%) at month 12, and 5 patients (0.4%) at month 18. Thus, 95% (1123/1177) of eligible patients had complete follow-up. There were no statistically significant differences between patients who completed the study and those who were lost to follow-up in terms of sex (*p* = 0.422) or age at inclusion (*p* = 0.340). Baseline characteristics of the study participants are summarized in Table 1 by fracture site.

**Table 1:** Baseline characteristics of participants by fracture site

Characteristics		Hip	Distal forearm	Vertebral	Humeral	Ankle
Number of fracture patients		223	237	183	166	214
Mean age, years (SD)		68.7 (9.8)	62.3 (8.1)	67.4 (8.7)	65.4 (9.3)	61.9 (7.9)
Women (%)		156 (70.0%)	205 (86.5%)	164 (89.6%)	131 (78.9%)	166 (77.6%)
Previous fractures in 5 years		54 (24.2%)	83 (35.0%)	131 (71.6%)	78 (47.0%)	108 (50.5%)
Hospitalization (%)		184 (82.5%)	47 (19.8%)	22 (12.0%)	84 (50.6%)	100 (46.7%)
Employed before fracture		33 (14.8%)	96 (40.5%)	38 (20.8%)	34 (20.5%)	78 (36.5%)
Full time job extent for employed		28 (84.9%)	89 (92.7%)	34 (89.5%)	30 (88.2%)	73 (93.6%)
Reason for not being employed	Old-age pensioner	171 (90.0%)	131 (92.9%)	102 (70.3%)	118 (89.4%)	120 (88.2%)
	Disabled pensioner	16 (8.4%)	6 (4.3%)	41 (28.3%)	10 (7.6%)	9 (6.6%)
Level of education	Primary	27 (12.1%)	13 (5.5%)	10 (5.5%)	6 (3.6%)	9 (4.2%)
	Secondary	127 (57.0%)	109 (46.0%)	54 (29.5%)	75 (45.2%)	79 (36.9%)
	University and professional diploma	68 (30.5%)	115 (48.5%)	119 (65.0%)	85 (51.2%)	126 (58.4%)
	Not reported	1 (0.5%)	0 (0%)	0 (0%)	0 (0%)	1 (0.5%)
Level of income	Low	82 (36.8%)	35 (14.8%)	29 (15.9%)	27 (16.3%)	22 (10.3%)
	Middle	125 (56.1%)	154 (65.0%)	128 (70.0%)	114 (68.7%)	151 (70.6%)
	High	10 (4.5%)	23 (9.7%)	24 (13.1%)	20 (12.1%)	30 (14.0%)
	Not reported	6 (2.7%)	25 (10.5%)	2 (1.1%)	5 (3.0%)	11 (5.1%)

Patients with wrist and ankle fractures were younger and were more frequently employed before the fracture. Low level of income was most frequent in hip fractures, and almost one in three vertebral fracture patients were disabled to the extent they could not work even before their index fracture. Previous fractures were most common in vertebral fracture subjects.

### Quality of life impairment after fracture

**Fig. 1:**
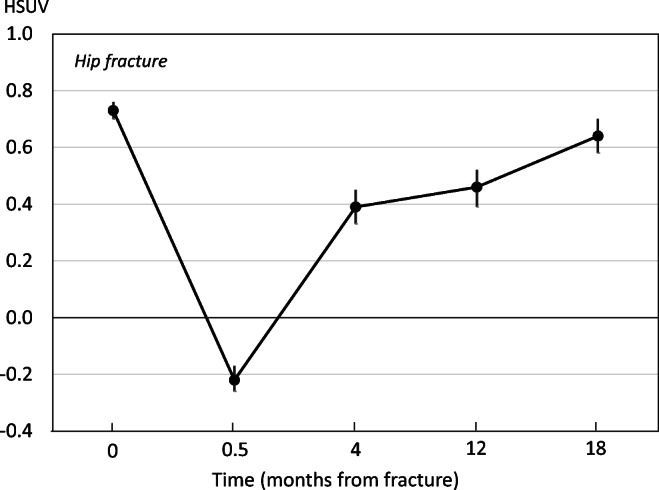
Mean health state utility value and 95% confidence intervals in patients with hip fracture before fracture, within 2 weeks after fracture, and at 4, 12, and 18 months after fracture

Quality of life from before fracture to 18 months after hip fracture is shown in Table 2 and Fig. 1. At all time-points, patients with hip fracture reported the lowest mean HSUV compared with other fracture sites. On average, hip fracture patients considered their HSUV to be worse than death within 2 weeks of fracture. Thereafter, utility values increased but remained significantly lower than before fracture, even at 18 months.

**Table 2:** Euroqol-5D mean health state utility value and 95% confidence intervals by fracture site before fracture, within 2 weeks after fracture, and at 4, 12, and 18 months after fracture

Fracture site	Before fracture		Within 2 weeks		At 4 months		At 12 months		At 18 months	
	Mean	95% CI	Mean	95% CI	Mean	95% CI	Mean	95% CI	Mean	95% CI
Hip	0.73	0.70–0.76	− 0.22	− 0.26,− 0.17	0.39	0.33–0.45	0.46	0.39–0.52	0.64	0.58–0.70
Distal forearm	0.90	0.88–0.92	0.46	0.42–0.49	0.83	0.80–0.86	0.86	0.84–0.89	0.89	0.87–0.92
Spine	0.80	0.77–0.83	0.24	0.19–0.30	0.69	0.64–0.73	0.73	0.69–0.77	0.70	00.66–0.75
Proximal humerus	0.85	0.82–0.88	0.29	0.23–0.25	0.70	0.66–0.74	0.73	0.70–0.77	0.77	0.74–0.80
Ankle	0.87	0.85–0.89	0.16	0.11–0.22	0.72	0.68–0.75	0.76	0.73–0.79	0.81	0.78–0.84

Patients with distal forearm fracture reported the highest mean HSUV throughout the period of observation. The decrement in utility values was less than for other fractures and had returned to pre-fracture levels by 18 months. For spine, humerus, and ankle fractures, there was a similar pattern of disutility with a nadir within 2 weeks and a progressive recovery thereafter. However, pre-fracture HSUV was not attained and values were significantly lower at 18 months (see Table 2).

The largest immediate QoL loss was observed in patients with hip fracture in whom mean immediate QoL loss was 0.94 (95% CI 0.90–0.98), followed by ankle fracture (0.71; 95% CI 0.66–0.76), humeral fracture (0.56; 95% CI 0.51–0.62), vertebral fracture (0.56; 95% CI 0.50–0.61), and distal forearm fracture (0.44; 95% CI 0.41–0.48).

Over 18 months after fracture, the accumulated mean QoL loss for hip, distal forearm, vertebral, humeral, and ankle fractures were estimated at 0.50 (95% CI 0.43–0.57), 0.13 (95% CI 0.11–0.17), 0.21 (95% CI 0.17–0.26), 0.26 (95% CI 0.21–0.31), and 0.27 (95% CI 0.22–0.32), respectively.

Mean bootstrapped QoL multipliers and corresponding 95% confidence intervals for 0–6, 0–12, and 12–18 months after fracture stratified by fracture type are given in Table 3. Mean bootstrapped QoL multipliers increased monotonically from 0 to 6 months to 12–18 months but remained significantly below 1.00 for all fracture sites 12–18 months after fracture.

**Table 3:** Mean bootstrapped QoL multiplier and 95% confidence interval within brackets stratified by fracture site and time after fracture

	0–6 months	0–12 months	12–18 months
Hip fracture	0.27 (0.22–0.33)	0.43 (0.37–0.50)	0.76 (0.68–0.82)
Distal forearm fracture	0.78 (0.76–0.81)	0.86 (0.84–0.88)	0.97 (0.95–1.00)
Vertebral fracture	0.68 (0.64–0.72)	0.79 (0.75–0.83)	0.89 (0.85–0.94)
Humeral fracture	0.66 (0.62–0.71)	0.76 (0.72–0.79)	0.89 (0.85–0.92)
Ankle fracture	0.62 (0.58–0.66)	0.74 (0.70–0.77)	0.91 (0.88–0.94)

### Recovery after fracture and predictors for recovery

**Fig. 2:**
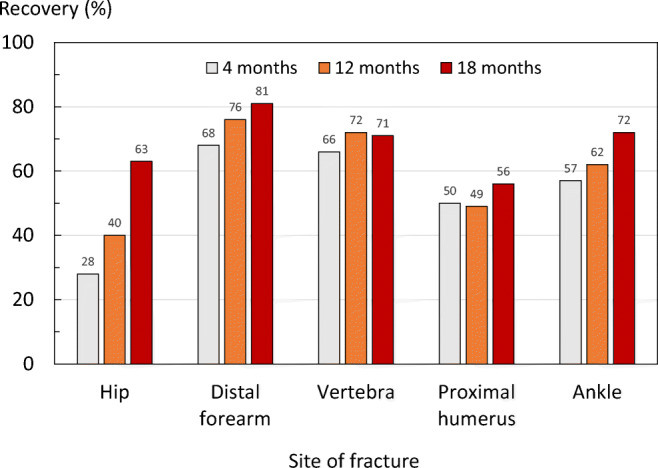
Number and proportion of patients who have recovered pre fracture HSUV at 4, 12, and 18 months after fracture, stratified by site

The proportion of patients who fully recovered 4, 12, and 18 months after fracture by site is illustrated in Fig. 2. Four months after fracture, patients who sustained a hip fracture had the lowest recovery rate (28%) followed by patients who sustained a humeral fracture (50%). Similarly, 12 months after fracture, patients who sustained a hip fracture had the lowest recovery rate (40%) followed by humeral fracture (49%). However, 18 months after fracture, the lowest recovery rate was observed in patients who sustained a humeral fracture (56%).

Table 4 shows the proportion of patients with severe impairment in each of the EQ-5D dimensions stratified by fracture type along with unadjusted and standardized differences in proportion of patients recovered at 18 months between patients with and without severe impairment. Patients with severe impairment in self-care and usual activities immediately after fracture had statistically significant lower probability of full recovery across all fracture types at 18 months in both unadjusted and standardized analyses. For self-care, point estimates of the unadjusted difference in probability of full recovery at 18 months ranged from 25% for hip and vertebral fracture to 57% for ankle fractures. For usual activities, point estimates of the difference in probability of full recovery at 18 months in crude analyses ranged from 22% for hip fracture to 48% for distal forearm and ankle fractures.

**Table 4:** Unadjusted and standardized absolute differences in probability of full recovery at 18 months in patients reporting severe impairment by EQ-5D dimension and fracture site

EQ-5D dimension	Patients with severe impairment in a given dimension immediately after fracture	Crude absolute difference in recovery rates between patients with severe impairment vs. no severe impairment	Standardized absolute difference in recovery rates between patients with severe impairment vs. no severe impairment
Hip fracture			
Mobility	123/149 (83%)	− 30%^b^	39%^c^
Self-care	106/149 (71%)	− 25%^b^	− 37%^c^
Usual activities	109/149 (73%)	− 22%^a^	− 27%^b^
Pain and discomfort	41/149 (28%)	16%	18%^a^
Anxiety depression	23/149 (15%)	0%	− 1%
Distal forearm fracture			
Mobility	20/174 (11%)	na	na
Self-care	26/174 (15%)	− 39%^c^	− 33%^a^
Usual activities	12/174 (7%)	− 48%^c^c	− 64%^c^
Pain and discomfort	4/174 (2%)	− 31%^b^	3%
Anxiety depression	0/174(0%)	− 81%^c^	na
Vertebral fracture			
Mobility	24/148 (16%)	− 23%^a^	− 24%^a^
Self-care	33/148 (22%)	− 25%^b^	− 23%^a^
Usual activities	47/148 (32%)	− 18%^b^	− 18%
Pain and discomfort	40/148 (27%)	− 15%	− 14%
Anxiety depression	8/148 (5%)	− 78%	na
Proximal humeral fracture			
Mobility	3/142 (2%)	14%	− 32%
Self-care	48/142 (34%)	− 48%	− 49%^c^
Usual activities	53/142 (37%)	− 48%	− 50%^c^
Pain and discomfort	37/142 (26%)	− 49%	− 50%^c^
Anxiety depression	24/142 (17%)	− 54%	− 53%^c^
Ankle fracture			
Mobility	88/192 (46%)	− 22%^c^	− 30%^c^
Self-care	40/192 (21%)	− 57%^c^	− 60%^c^
Usual activities	67/192 (35%)	− 45%^c^	− 50%^c^
Pain and discomfort	44/192 (23%)	− 11%	− 20%^a^
Anxiety depression	13/192 (7%)	− 53%^c^	− 48%^b^

^a^*p* < 0.05, ^b^*p* < 0.01, ^c^*p* < 0.001. No patient reported severe mobility impairment after distal forearm fracture and therefore no differences in recovery rates could be computed for severe mobility impairment (yes/no) after distal forearm fracture. No standardized differences in absolute recovery probabilities could be computed for severe anxiety/depression after distal forearm fracture and vertebral fracture

## Discussion

This study presents data on the QoL impact of hip, vertebral, proximal humerus, distal forearm, and ankle fracture up to 18 months after fracture from the Russian arm of the ICUROS study. The information obtained in current study is of particular importance since existing data on QoL related to osteoporotic fractures in Russia are scarce.

The accumulated mean QoL loss after hip fracture was 0.5 over 18 months and significantly greater than after fracture of the distal forearm (0.13), spine (0.21), proximal humerus (0.26), and ankle (0.27). The very marked utility loss after hip fracture and partial recovery is very consistent with pattern identified in systematic reviews of studies from other countries that used EQ-5D for QoL assessment [7, 17], as well as in other ICUROS countries [9, 18, 19]. Patients with distal forearm fracture on average regained pre-fracture QoL at 18 months after fracture as reported elsewhere [7, 8], whereas patients with humeral, ankle, and vertebral fracture experienced sizable decrements in HSUVs of approximately 0.10 compared with before the fracture. Substantial impairment in self-care and usual activities immediately after fracture were important predictors of recovery across at all fracture sites and the effects generally remained after controlling for age, sex, and VAS prior to fracture. For example, among patients with a proximal humeral fracture, the absolute difference in recovery rates between the 67/192 (35%) of patients who reported severe impairment in usual activities immediately after fracture and the remaining 125/192 (65%) patients were 60%.

QoL in patients with humeral fragility fractures of the proximal humerus has been less well documented than that following forearm, spine, or hip fracture [20, 21]. In the present study, the pattern of response and cumulative disutility was similar to that after vertebral fracture. Interestingly, the data from the Australian ICUROS showed that the decline in HRQoL for humeral fractures was even greater in the immediate fracture period than for vertebral fractures returned to pre-fracture levels by 18 months [18].

Information on HSUVs following ankle fractures are scarce and reported previously only in the Australian arm of ICUROS [18]. The reason is that ankle fractures are not considered to be characteristic of osteoporosis [22]. Fractures of the ankle are inconsistently associated with low BMD in elderly women [23, 24]. There is, moreover, no age-related increase in risk from the age of 50 years in men or in women [22]. It is relevant that the risk factors for ankle fractures in women after the menopause differ from those for other osteoporotic fractures. For example, high body weight, but not early menopause are risk factors for ankle fractures, whereas low body weight and early menopause are risk factors for wrist fractures [25, 26]. The pattern of change in HSUVs after ankle fracture was similar to that of spine and humerus fracture as was the mean cumulative utility loss.

Despite qualitative similarities between studies, there are a number of quantitative differences between studies and countries. The disparities in short-term QoL multipliers may reflect time to interview after first fracture: longer time to interview may result in lower initial QoL decrement. In Russia, not all hip fracture patients are surgically managed and may contribute to the very high utility loss within 2 weeks after hip fracture. Over the longer term, there are also substantial differences. For example, compared with Russia, the accumulated QoL losses over 18 months were higher in Italy and Lithuania and lower in Austria for hip, humeral, and distal forearm fracture in the ICUROS study where data collection was standardized [4]. Despite standardization, the severity of fractures may differ between the studies depending on the participating centers. Some centers may be highly specialized and therefore treat more severe fractures or more frail patients than others. There will also be a consent bias, particularly in the case of hip fracture, in that many patients who sustain a hip fracture will also have dementia and would be excluded from study. The method of recruitment is of critical importance for vertebral fracture. In the Australian arm of ICUROS, the majority of patients with vertebral fracture were recruited through an emergency department [18]. In the Russian arm, participants were recruited mainly from Neurology or Radiology Departments, several days or weeks after the fracture event. Also, HRQoL loss varies according to the number and severity of vertebral fracture [27], which was not taken into account in ICUROS. The mean age of hip fracture in the present study (69 years) was less than in the other ICUROS countries (76 years) [8]. This is likely a reflection of the higher general population mortality in Russia but limits the interpretation to a younger population than in other ICUROS countries. For all these reasons, between-center and between-country differences in HSUVs are difficult to interpret. Notwithstanding, a persistent disutility at 18 months is a consistent finding for hip [28–30] and spine fractures [8, 31]. Conversely, there is general agreement that the disutility following fracture of the distal forearm is transient [32, 33]. In ICUROS, recovery was on average incomplete before 18 months which contrasts with a much more rapid recovery pattern, albeit on fewer patients from the UK [33].

There are several additional limitations of ICUROS study [8, 18, 34]. HSUV prior to fracture was determined by recall with a potential for bias. However, it has been shown that patients can accurately recall their QoL up to 6 weeks [35], so that substantial recall bias is unlikely. This assumption is supported by the finding in patients with distal forearm fractures that HSUV 18 months after distal forearm fracture was similar to mean HSUV prior to fracture, consistent with modest and, on average, a transient natural history [32]. The exclusion criteria (long-term care prior to the fracture, cognitive impairment, and patients sustaining a new fracture during follow-up) are likely to bias the HSUVs and account also for the low mortality in this cohort. For this reason, the recovery rates may be better than average recovery rates in the general population.

In ICUROS, approximately 21% of patients were lost to follow-up. In the present study, the dropout rate was substantially less (8.3%). Rather than lessen bias, the low dropout rate, particularly for mortality (1.7%), reinforces the notion of preferential recruitment of a healthier segment of the population.

In conclusion, this study shows that fractures of the hip, vertebral, distal forearm, ankle, and proximal humerus incur substantial loss of QoL in Russia. Although there is marked variation in QoL losses between fracture sites, and with the exception of forearm fractures, QoL is markedly impaired for at least 18 months. Furthermore, severe impairment in self-care or usual activities after fracture indicate that patients may have suboptimal long-term recovery. In such studies, these results can help inform health technology assessment and early identification of patients with poor long-term prognosis, specifically for Russia.
